# Trauma team activation varies across Dutch emergency departments: a national survey

**DOI:** 10.1186/s13049-015-0185-0

**Published:** 2015-11-16

**Authors:** Rolf E. Egberink, Harm-Jan Otten, Maarten J. IJzerman, Arie B. van Vugt, Carine J. M. Doggen

**Affiliations:** Acute Zorg Euregio, PO Box 50.000, 7500 KA Enschede, The Netherlands; Department of Health Technology and Services Research, MIRA Institute for Biomedical Technology and Technical Medicine, University of Twente, PO Box 217, 7500 AE Enschede, The Netherlands; Emergency Department, Medisch Spectrum Twente, PO Box 50.000, 7500 KA Enschede, The Netherlands

**Keywords:** Emergency medical services, Emergency service hospital, Emergency nursing, Multiple trauma, Triage, Patient care team, Decision making, Emergency department, In-hospital trauma triage, Trauma team activation

## Abstract

**Background:**

Tiered trauma team response may contribute to efficient in-hospital trauma triage by reducing the amount of resources required and by improving health outcomes. This study evaluates current practice of trauma team activation (TTA) in Dutch emergency departments (EDs).

**Methods:**

A survey was conducted among managers of all 102 EDs in the Netherlands, using a semi-structured online questionnaire.

**Results:**

Seventy-two questionnaires were analysed. Most EDs use a one-team system (68 %). EDs with a tiered-response receive more multi trauma patients (*p* < 0.01) and have more trauma team alerts per year (*p* < 0.05) than one-team EDs. The number of trauma team members varies from three to 16 professionals. The ED nurse usually receives the pre-notification (97 %), whereas the decision to activate a team is made by an ED nurse (46 %), ED physician (30 %), by multiple professionals (20 %) or other (4 %). Information in the pre-notification mostly used for trauma team activation are Airway-Breathing-Circulation (87 %), Glasgow Coma Score (90 %), and Revised Trauma Score (85 %) or Paediatric Trauma Score (86 %). However, this information is only available for 75 % of the patients or less. Only 56 % of the respondents were satisfied with their current in-hospital trauma triage system.

**Conclusions:**

Trauma team activation varies across Dutch EDs and there is room for improvement in the trauma triage system used, size of the teams and the professionals involved. More direct communication and more uniform criteria could be used to efficiently and safely activate a specific trauma team. Therefore, the implementation of a revised national consensus guideline is recommended.

**Electronic supplementary material:**

The online version of this article (doi:10.1186/s13049-015-0185-0) contains supplementary material, which is available to authorized users.

## Background

### Trauma team activation

The activation of a multidisciplinary trauma team to assess and treat seriously injured patients is an integral part of the management of trauma and has been shown to improve health outcomes [1–5]. Since the introduction of trauma teams, most emergency departments (EDs) worldwide use a one-team trauma response: one type of trauma team is activated for every incoming trauma patient. Due to growing concerns of overtriage and increasing costs, tiered-response trauma team activation (TTA) was introduced in the nineties [4, 6–15]. If a one-team response is used, a full trauma team is required in the ED for every trauma patient. When using a tiered-response, the size and expertise of a trauma team is tailored to the condition of the patient: activation of a full trauma team for severely injured patients and a modified trauma team for patients with minor injuries [3–5]. Activating a modified team means using less staff and resources, and therefore also results in less disruption of other clinical activities elsewhere in the hospital. The decision to activate a specific team usually is an in-hospital triage decision and is mostly guided by protocols or algorithms using multiple criteria and scoring systems [1–8, 10, 13, 14, 16].

In-hospital trauma triage systems have particularly focussed on the appropriate use of resources within the hospital [6]. Triage criteria for TTA must balance the required resources to provide care and the probability of undertriage: under-treatment resulting in avoidable morbidity or mortality [1]. Overtriage obviously is costly and may be considered an inefficient use of staff and resources [6, 14]. Internationally, size and composition of trauma teams and the process and criteria for in-hospital TTA are reported to vary on a local basis depending on resources, experience and level of adoption [2, 4, 5, 14, 17–21]. A system with a tiered-response has been shown to contribute to a safe and efficient TTA by reducing the amount of resources required and by improving patient outcomes, but may be inappropriate in EDs with a low number of trauma patients or with less experience in trauma care [4, 6–8, 10–14, 16].

### Trauma teams in the Netherlands

Since 1997 trauma care in the Netherlands has been part of eleven regional healthcare systems, incorporating regional organisation of individual Emergency Medical Services (EMS), the designation of trauma centres, and the creation of a national network of Helicopter Emergency Medical Services (HEMS) [22]. Hospitals with an ED are classified in three levels of care facilities, namely: level 1 hospitals with full facilities (trauma centres) providing multi trauma care; level 2 hospitals with intermediate facilities for trauma without the need for neurosurgery; and, level 3 hospitals with basic facilities for the care of a trauma patient [1, 22]. Allocation of trauma patients to a hospital with the appropriate level of facilities is organized according to a pre-hospital trauma triage flowchart from the Dutch national protocol for EMS [6, 22–24]. ED staff is preferably pre-notified of an incoming trauma patient, directly by the (H)EMS team, or indirectly by an Emergency Medical Dispatcher (EMD), and activates a trauma team according to the hospital protocol [6].

In the Netherlands, an algorithm is available from the Dutch national protocol for the ED on when to upgrade the standard ED team and recommendations for communication of the pre-hospital information and the size and composition of Dutch trauma teams are available in national EMS and ED guidelines and standards for trauma surgeons [23, 25, 26]. It is unknown to what extent these protocols and guidelines are used in practice and if uniform criteria are being used in the different EDs.

In 2010 concentration of high complex patients, such as multi trauma patients, took place in the Netherlands. The number of multiply injured patients presented to level 2 and 3 EDs has reduced since then [22, 27]. The infrequency of trauma patients in these EDs should not deter the formation of trauma teams, in fact it is a major reason for forming such teams [18]. Only one study described the use of tiered trauma team response in the Netherlands [6]. No information on the national level on TTA is available. The primary objective of the present study therefore is to evaluate the existing practice of TTA in EDs in the Netherlands. More specifically, we investigated size and composition of different teams, communication and decision making using patient information, and satisfaction with the current system, comparing EDs with a one-team and with a tiered-response in-hospital trauma triage system. Results might facilitate the implementation of a revised national consensus or guideline that contributes to a safe and efficient deployment of trauma teams in the Netherlands.

## Methods

### Study design and population

A semi-structured online questionnaire was sent out to all ED managers of Dutch hospitals providing trauma care. ED managers were identified using a list of 102 Dutch hospitals with an ED [28]. A brief explanation of the objectives of the study was given using a telephone call. ED managers that wished to participate received an e-mail with further explanation of the study and a hyperlink to fill out the questionnaire online. Reminders were sent after four weeks. Questionnaires were filled out between May 30, and July 26, 2011. The study was submitted to the regional medical ethics committee and was deemed exempt from ethical review according to Dutch law governing scientific research with humans.

### Questionnaire

The questionnaire consisted of the following topics: general characteristics of the ED (including number of multi trauma patients and number of trauma team alerts per year), type of in-hospital trauma triage system, composition of different trauma teams, type of communication between ED and (H)EMS, available information from the pre-hospital setting, criteria used in the decision making process at the ED, satisfaction and usefulness of currently used trauma triage system.

The items in the questionnaire were selected based on a review of the literature on in-hospital trauma triage, supplemented with information obtained through participant observation at a level 1 trauma centre and a regional ambulance service. The questionnaire was further developed and evaluated for content and readability by an expert panel, consisting of an ED physician, trauma surgeon, ED manager, epidemiologist and a policy advisor on trauma care.

### Statistical analysis

Data were analysed using descriptive statistics. Results are presented in frequencies, percentages, medians and range (min-max). Counts for different categories were compared by the Chi-square test. Statistics were performed with the use of SPSS version 18 (SPSS Inc., Chicago, IL).

## Results

Ninety out of 102 (88 %) ED managers responded to the online questionnaire. Thirteen questionnaires were excluded because less than 50 % of the questions were answered and another five were excluded because no formalized trauma team was used in the ED. This resulted in 72 (71 %) questionnaires used for analysis. Eleven of the 13 level 1 EDs in the Netherlands participated (85 %), 32 of the 45 level 2 (71 %) and 29 of the 44 level 3 EDs (66 %). See Additional file 1: Table S1a, which presents characteristics of the participating EDs by in-hospital trauma triage system used.

### In-hospital trauma triage systems in practice

Most EDs use a one-team trauma triage system (*n* = 49, 68 %), 23 EDs (32 %) use a tiered trauma response system, with either two or three teams (Table 1). EDs using a one-team trauma triage system can be level 1, 2 or 3 EDs. Most of these EDs receive less than 50 multi trauma patients (80 %) and have less than 50 trauma team alerts (80 %) each year. More than 50 % of the one-team EDs are intermediate in size, according to the total number of ED patients per year (10,000 to 25,000) and number of full-time equivalent (FTE) emergency nurses (15 to 30 FTE). EDs using a tiered-response are mostly level 1 and 2 EDs. The EDs with a tiered-response receive more multi trauma patients annually (*p* < 0.01), have more trauma team alerts per year (*p* < 0.05) and are larger than the one-team EDs, according to the total number of ED patients per year and number of FTE emergency nurses (Table 1).

**Table 1:** Characteristics of EDs

	One team	Tiered response	*P*-value*
	*n* = 49	*n* = 23	
	(68.1 %)	(31.9 %)	
Level of ED, *n* (%)			0.05
Level 1 (*n* = 11)	5 (10.2)	6 (26.1)	
Level 2 (*n* = 32)	20 (40.8)	12 (52.2)	
Level 3 (*n* = 29)	24 (49.0)	5 (21.7)	
Number of multi trauma patients per year, *n* (%)			<0.01
<50 (*n* = 51)	39 (79.6)	12 (52.2)	
50-200 (*n* = 12)	8 (16.3)	4 (17.4)	
>200 (*n* = 9)	2 (4.1)	7 (30.4)	
Number of trauma team alerts per year, *n* (%)			<0.05
<50 (*n* = 51)	39 (79.6)	12 (52.2)	
50-200 (*n* = 13)	8 (16.3)	5 (21.7)	
>200 (*n* = 8)	2 (4.1)	6 (26.1)	
Total number of ED patients per year, *n* (%)			0.29
<10,000 (*n* = 8)	7 (14.3)	1 (4.3)	
10,000-25,000 (*n* = 40)	28 (57.1)	12 (52.2)	
>25,000 (*n* = 24)	14 (28.6)	10 (43.5)	
FTE ED nurses, *n* (%)			0.59
<15 (*n* = 10)	8 (16.3)	2 (8.7)	
15-30 (*n* = 44)	30 (61.2)	14 (60.9)	
>30 (*n* = 18)	11 (22.4)	7 (30.4)	

*ED* Emergency Department, *FTE* Full-Time Equivalent

*Chi-square test

### Size and composition of trauma teams

The overall number of trauma team members varies from three to 16 professionals from different medical, nursing and other health specialties (see Additional file 1: Table S1b, which illustrates the composition of the different trauma teams by in-hospital trauma triage systems used). Trauma teams in a one-team ED have a median of seven team members (three to 13 team members). Of the 23 EDs with a tiered-response 19 have two teams and four EDs have three teams. The median number of team members for the largest team is ten (five to 16 team members), eight for the intermediate team (five to 12 team members) and for the smallest team five (three to seven team members). Composition of the different teams varies widely, but more than 50 % of the one-team teams and the largest teams of tiered-response EDs consist of at least two ED nurses, an emergency physician, a (trauma)surgeon, a surgery/orthopaedics resident, an anaesthesiologist, a radiologist and a radiographer. More than 50 % of the smallest teams of tiered-response EDs consist of at least one ED nurse, an emergency physician, a surgery/orthopaedics resident and a radiographer. Some EDs describe the possibility to activate specific additional staff next to the formalized composition of the various teams, dependent on the needs of the patient, e.g. a thoracic surgeon.

### Communication and decision making using patient information

Sixty-nine of the 72 EDs (96 %) receive a pre-notification from (H)EMS about the incoming trauma patient, mostly by telephone (93 %) (Table 2). Of the pre-notifications 39 % is communicated directly by (H)EMS to ED, 32 % indirectly to the ED through an EMD and 20 % by both (H)EMS and EMD. The ED nurse usually receives the pre-notification (97 %). There were no differences between the one-team and tiered-response EDs with regard to the professional communicating the pre-notification (Table 2). The decision for TTA is made by an ED nurse (46 %; of which 20 % by the ED nurse receiving the pre-notification), ED physician (30 %), or by multiple professionals (20 %). In EDs with a tiered-response nurses more often appeared to decide about TTA compared to one-team EDs (59 % versus 40 %). ED physicians make less decisions on TTA in tiered-response EDs compared to one-team EDs (18 % versus 36 %). However, the differences in the professional making the decision was not significantly different (*p* = 0.29).

**Table 2:** Communication and decision making using patient information

	Total	One team	Tiered response	*P*-value**
	*n* = 69*	*n* = 47	*n* = 22	
	*n* (%)	*n* (%)	*n* (%)	
Type of pre-notification				0.28
Telephone call	64 (92.8)	42 (89.4)	22 (100.0)	
Digital on mobile device	1 (1.4)	1 (2.4)	0 (0.0)	
Other^a^	4 (5.8)	4 (8.5)	0 (0.0)	
Professional sending pre-notification from pre-hospital setting				0.94
EMD (indirect)	22 (31.9)	15 (31.9)	7 (31.8)	
(H)EMS (direct)	27 (39.1)	19 (40.4)	8 (36.4)	
(H)EMS and EMD (direct and indirect)	14 (20.3)	9 (19.1)	5 (22.7)	
Other^b^	5 (7.2)	3 (6.4)	2 (9.1)	
Unknown	1 (1.4)	1 (2.1)	0 (0.0)	
Professional receiving pre-notification at ED				0.27
ED nurse	67 (97.1)	46 (97.9)	21 (95.5)	
first ED nurse present	39 (56.5)	26 (55.3)	13 (59.1)	
triage nurse	17 (24.6)	14 (29.8)	3 (13.6)	
coordinator/senior nurse	11 (15.9)	6 (12.8)	5 (22.7)	
ED physician	1 (1.4)	0 (0.0)	1 (4.5)	
Other^c^	1 (1.4)	1 (2.1)	0 (0.0)	
Professional making decision for TTA				0.29
ED nurse	32 (46.4)	19 (40.4)	13 (59.1)	
ED nurse receiving pre-notification	14 (20.3)	9 (19.1)	5 (22.7)	
triage nurse	10 (14.5)	5 (10.6)	5 (22.7)	
coordinator/senior nurse	8 (11.6)	5 (10.6)	3 (13.6)	
ED physician	21 (30.4)	17 (36.2)	4 (18.2)	
emergency physician	11 (15.9)	8 (17.0)	3 (13.6)	
(trauma) surgeon	8 (11.6)	7 (14.9)	1 (4.5)	
other physician (not specified)	2 (2.9)	2 (4.3)	0 (0.0)	
Multiple professionals	14 (20.3)	9 (19.1)	5 (22.7)	
ED nurse and EMS	1 (1.4)	1 (2.1)	0 (0.0)	
ED nurse and emergency physician	6 (8.7)	4 (8.5)	2 (9.1)	
meeting with whole ED team	7 (10.1)	4 (8.5)	3 (13.6)	
Other^d^	2 (2.9)	2 (4.3)	0 (0.0)	

*ED* Emergency Department, *EMD* Emergency Medical Dispatcher, *EMS* Emergency Medical Service, *HEMS* Helicopter Emergency Medical Services, *TTA* Trauma Team Activation

*Three of the EDs do not receive pre-notification; at these EDs decision for TTA is made by an ED nurse (coordinator/senior nurse) and other (*n* = 2; ED floor manager and protocol)

**Counts for different categories were compared by the Chi-square test

^a^By telephone call and electronically (*n* = 3), only electronically (screen on ED)

^b^Multiple possibilities (*n* = 2), electronically, mostly by EMS nurse and sometimes by EMD

^c^ED nurse or emergency physician or secretary

^d^Not specified

The information most frequently available in a pre-notification is: blood pressure (83 %), pulse rate (80 %), and age and gender (both 77 %) (Table 3). However, other less available (<75 %) parameters are most often used as a criterion for TTA: Airway-Breathing-Circulation (87 %), Glasgow Coma Score (90 %) and the Revised Trauma Score (85 %) or Paediatric Trauma Score (86 %). Fifty-four of the 72 EDs (75 %) have a protocol for in-hospital trauma triage. EDs with a tiered response more often have a protocol than EDs with one team.

**Table 3:** Availability of information in pre-notification (*n* = 69) and use in ED as a criterion for TTA

	Available in pre-notification	Of which used as a criterion
	*n* (%)	*n* (%)*
Demographic information		
Age	53 (76.8)	22 (41.5)
Gender	53 (76.8)	5 (9.4)
Pregnancy	37 (53.6)	21 (56.8)
Mechanism of Injury information	51 (73.9)	41 (80.4)
Physiologic parameters		
Respiratory rate	45 (65.2)	27 (60.0)
Oxygen saturation	49 (71.0)	26 (53.1)
Pulse rate	55 (79.9)	29 (52.7)
Blood pressure	57 (82.6)	30 (52.6)
Airway-Breathing-Circulation	52 (75.4)	45 (86.5)
Glasgow Coma Score	48 (69.9)	43 (89.6)
Body temperature	18 (26.1)	11 (61.1)
Revised Trauma Score	39 (56.5)	33 (84.6)
Paediatric Trauma Score	28 (40.6)	24 (85.7)
Treatment given	48 (69.6)	33 (68.8)
Other information		
Medical history	25 (36.2)	9 (36.0)
Infectious diseases	20 (29.0)	4 (20.0)

*ED* Emergency Department, *TTA* Trauma Team Activation

*Percentage was calculated dividing number of used criteria by number of available criteria

### Overall usefulness

Of the 72 respondents 56 % were satisfied with the current situation on in-hospital trauma triage and found their system useful. Satisfaction was higher in EDs with a tiered-response (65 % versus 53 %) and in EDs with a protocol present (62 % versus 44 %). Twenty-five respondents gave suggestions for improvement, including improvements for the ED or trauma care in general. Nine suggestions concern the communication between the pre-hospital setting and the ED, e.g. better use of the (electronic) pre-notification. Twelve of the 18 EDs without a protocol stated that a protocol with clear criteria for TTA would be an improvement for the whole trauma triage process.

## Discussion

This study found that most Dutch EDs use a one-team trauma triage system, although a large variation was found in size and composition of trauma teams and the TTA process. Our results are largely consistent with the local variation found in several international studies on TTA [2, 4, 5, 14, 17–21]. Nonetheless, this is somewhat surprising considering that the quality improvement activities for trauma care in the Netherlands since 1997 [22, 24] and the existence of national guidelines [23, 25, 26, 29] should suggest a more uniform TTA.

### In-hospital trauma triage systems in practice

In the Netherlands, 94 % of the responding EDs indicate the presence of a trauma team, compared to 21 % to 98 % in other countries [12, 14, 15, 17, 18, 30]. Moreover, two-thirds of Dutch EDs use a one-team system. The relative low percentage of hospitals using tiered-response system is surprising, because previous studies have shown that a tiered-response is a safe and effective trauma triage method. Efficiency may also be improved because undertriage does not exist and overtriage has decreased from 70 % to 27 % [6]. In the latter study the in-hospital trauma triage was performed only after arrival of the patient, implying that for every trauma patient a full team was activated initially. Although tiered-response may be efficient, its benefits will not be reported if it is used after pre-notification of a trauma patient. Tiered-response after arrival of the patient also introduces delays in the management of the severely injured patient and is insufficiently sensitive to prevent under-activation of the team [9]. In our study, two EDs with more than 200 trauma team alerts, and another eight with 50 to 200 trauma team alerts per year used a one-team response. These relatively large EDs could possibly benefit from introducing a tiered-response system. We also found 12 EDs receiving less than 50 multi trauma patients per year and with less than 50 trauma team alerts per year using a tiered-response system. These EDs could probably best use a one-team system to maintain sufficient expertise and quality of trauma care. The overtriage in these cases then provides a training-opportunity [15].

### Required size and expertise of trauma teams

In general, the optimum size of a trauma team varies from five (modified trauma team) to eight (full trauma team) team members [1, 2, 5, 31]. However, this study showed that Dutch trauma teams varied from three to 16 members. Internationally, the size of trauma teams also varies but less widely, i.e. from two to ten team members [2, 4, 5, 8, 14, 17–21]. Based on these figures, this study suggests that a reduction in the number of trauma team members is possible. Such reduction may lead to a cost-saving of at least $431 in indirect cost savings per trauma team activation [10].

In addition to the size of the team the expertise required in the trauma team is vital to its function and efficiency [5]. The vast majority of full trauma teams in the Netherlands consists of a minimum required expertise that is close to what is observed in other countries [1, 2, 5, 14, 18]. The only deviation is the presence of a neurologist which is recommended according to the Dutch guidelines, yet only found in 31 % of the EDs in our study [22, 25, 26]. In other countries the neurological status of a trauma patient often is assessed by an emergency physician, who is not yet 24/7 present in all Dutch EDs [27, 32]. Our study shows that the smallest of the modified trauma teams have an emergency physician and/or a surgical or orthopaedic resident, a radiographer and one ED nurse present. Whether this corresponds to the optimum composition of a modified team is not known, recommendations from scientific studies or Dutch guidelines are not available.

The ideal team should at least be available at all hours and consists of appropriately trained staff, such that junior members are not left alone in dealing with trauma cases [5, 14, 31, 33]. Surprisingly, the minimum level of expertise for full trauma teams differs between office-hours and out-of-office-hours [26], even though 47 % of all trauma patients arrive at the ED between 5 PM and 8 AM [34]. Therefore, future research should address the optimum composition of (modified) trauma teams, keeping in mind that commitment, organisation, training, experience and 24/7 availability are found to be more important than specialty or seniority of the individual professionals [2, 5, 14, 31, 33].

To contribute to a reduction of team size, the standard initial involvement of a neurologist, orthopaedic surgeon, ICU physician, neurosurgeon, and paediatrician could be reconsidered, especially since their participation in Dutch trauma teams is already limited and emergency physicians are being introduced as a 24/7 senior specialist in Dutch EDs. This professional could well play a more important role in initial trauma team care in the near future [27, 32], as they do in several countries [14, 30].

### Communication and decision making using patient information

For a timely activation and preparation of an appropriate trauma team, ED staff need accurate information from the pre-hospital setting, preferably before patient’s arrival [1–5, 14, 16]. To avoid errors, such pre-notification should ideally be communicated directly from the pre-hospital caregiver to the trauma triage decision maker in the hospital. Our study shows that the pre-notification is communicated indirectly through an emergency medical dispatcher in 32 % of the EDs, and the decision on TTA is not made by the same person who receives the information initially.

In several countries, the decision on TTA was mostly found to be a medical staff decision and not a decision made by ED nursing staff, except in some EDs in the US and Australia [3, 7, 11]. ED nurses in the Netherlands are well trained, are 24/7 present in the ED [22, 24] and this study shows they receive the pre-notification in 97 % of the EDs. Nevertheless, it is not clear if ED nurses are the best suited professionals to make the decision on TTA.

Internationally, most EDs use a combination of anatomical, physiological and mechanistic criteria to activate a trauma team. However, none are universally accepted and criteria vary on a local or regional basis [2, 3, 9, 11, 14–16, 18]. Our results also show a wide variation in the criteria used by the ED staff to activate their trauma team. Variability in TTA criteria within the same country and region likely depends on the available resources and personnel responsible for setting up the trauma system, and are guided by a combination of experience from other hospitals, local adjustments and expert opinion, rather than based on an analysis of the trauma population [13, 15, 16]. TTA criteria should be a result from an analysis of in-hospital triage performed using the local or regional trauma population to achieve desired sensitivity [3, 5, 7, 13, 16].

Improved standardisation of activation criteria across EDs is needed. Advantages in standardization of criteria in a region or country with comparable trauma populations include: compatibility with EMS protocols improving communication; optimization of patient care; training; enabling staff-rotation between EDs; research and audit [16]. In addition, it is questionable whether the widespread use of the US ACS-COT field triage criteria for TTA [1, 15] is suitable for in-hospital use in other (European) countries. Despite the fact that the ACS-COT already deleted the Revised Trauma Score in 2006 [1, 35] as a triage criterion, it is still present in Dutch guidelines [23, 25] and when available to the ED (57 %) used in 85 % as a criterion for TTA.

Because of the existence of national guidelines [23, 25, 26, 29] a more uniform TTA was expected. Unfortunately, a study on the use of the Dutch national ED protocol also shows poor adherence (38 %) [36] and the recently developed Netherlands Triage Standard, for use in the ED, general practitioners and EMS, contains no algorithm for TTA [27, 29]. With the introduction of a new version of the national EMS protocol in 2014, the methodology for communication of pre-hospital information to the hospital changes [37]. Guidelines, training, local protocols and registration systems should be aligned with this pre-hospital protocol.

### Strengths and limitations

By using online questionnaires, we were able to obtain an overview of nationwide clinical practice on this specific subject, in a relatively quick way with relatively low cost. By inviting all Dutch EDs to participate in this first national survey we were able to analyse the mechanisms for possible differences in in-hospital trauma triage between and within the different types of EDs in the Netherlands. With a high response rate and the participation of EDs at all levels of trauma care from all over the country, the results are representative for the situation on TTA in the Netherlands. Unfortunately 13 questionnaires of initial responders had to be excluded because less than 50 % of the questions were answered, possibly due to the length of the questionnaire. As this is likely to be a random event we do not expect this to have influenced our results. Due to registration shortcomings, most EDs could not provide exact numbers on trauma team alerts and therefore these numbers were probably estimated close to the number of multi trauma patients. In practice EDs probably have more trauma team alerts than multi trauma patients and therefore even more EDs could probably benefit from a tiered-response trauma triage system.

## Conclusions

In contrast to other countries, the concept of a trauma team is adopted by the majority of Dutch EDs involved in trauma care. Nevertheless there is room for improvement on several aspects: 1. Large EDs may benefit from introducing a tiered-response system and intermediate to small EDs may be better off using a one-team system; 2. Size and composition of trauma teams varies more widely than reported in other countries and a reduction of trauma team members may be possible and cost-saving; 3. Information that is used for in-hospital decision making could be communicated directly from pre-hospital caregiver to the decision maker, however, it is uncertain which professional is the best suited to make the decision on TTA; 4. The variety in TTA criteria employed and the in-hospital trauma triage process suggests the need for a more uniform set of criteria that could be used to efficiently and safely activate a specific trauma team. To address all aspects mentioned above, we recommend additional research which facilitates the development of a decision support instrument and secondly, the implementation of a revised national or European consensus guideline that contributes to a safe and efficient deployment of trauma teams.

## Additional file

Additional file 1:
**Table S1a: Characteristics of the participating EDs by in-hospital trauma triage systems used Table S1b: Composition of the different trauma teams by in-hospital trauma triage systems used.** (PDF 31 kb)
