# Supplementary material for: Trauma team activation varies across Dutch emergency departments: a national survey
Source: Scand J Trauma Resusc Emerg Med. 2015 Nov 16;23:100. doi: 10.1186/s13049-015-0185-0 (PMC4647827; doi:10.1186/s13049-015-0185-0)
Supplement: Additional file 1: — Table S1a: Characteristics of the participating EDs by in-hospital trauma triage systems used Table S1b: Composition of the different trauma teams by in-hospital trauma triage systems used. (PDF 31 kb) [file 13049_2015_185_MOESM1_ESM.pdf]

Trauma team activation varies across Dutch emergency departments: a national survey
Rolf E. Egberink et al.

Additional file 1

Table S1a. Characteristics of emergency departments by level of trauma care (over the year 2010)

	**Total** n = 72	**Level 1** n = 11	**Level 2** n = 32	**Level 3** n = 29
Multi trauma patients per year, n (%)				
< 50	51 (70.8)	0 (0.0)	25 (78.1)	26 (89.7)
50-200	12 (16.7)	5 (45.5)	4 (12.5)	3 (10.3)
> 200	9 (12.5)	6 (54.5)	3 (9.4)	0 (0.0)
Team alerts per year, n (%)				
< 50	51 (70.8)	0 (0.0)	23 (71.9)	28 (96.6)
50-200	13 (18.1)	5 (45.5)	7 (21.9)	1 (3.4)
> 200	8 (11.1)	6 (54.5)	2 (6.2)	0 (0.0)
ED patients per year, median (min-max)	22,192 (2,639-49,681)	30,224 (22,384-49,681)	24,081 (10,500-46,794)	14,500 (2,639-28,484)
FTE ED nurses, median (min-max)	23 (9-59)	38 (24-59)	23 (13-52)	19 (9-48)

ED = Emergency Department; FTE = Full-Time Equivalent

Table S1b. Composition of the different trauma teams by in-hospital trauma triage systems used

	**One team** n=47*	**Tiered response** n=23**		
		Large team n=23	Intermediate team n=4	Small team n=23
Number of team members, median (min-max)	7 (3-13)	10 (5-16)	7.5 (5-12)	5 (3-7)
	n (%)	n (%)	n (%)	n (%)
Nursing staff				
ED nurse #1	45 (95.7)	23 (100.0)	4 (17.4)	23 (100.0)
ED nurse #2	38 (80.9)	22 (95.7)	4 (17.4)	11 (47.8)
ED nurse #3	4 (8.5)	0 (0.0)	0 (0.0)	1 (4.3)
Medical staff				
emergency physician	28 (59.6)	17 (73.9)	4 (17.4)	17 (73.9)
resident surgery/orthopaedics #1	37 (78.7)	21 (91.3)	4 (17.4)	21 (91.3)
resident surgery/orthopaedics #2	3 (6.4)	5 (21.7)	1 (4.3)	3 (13.0)
(trauma) surgeon	37 (78.7)	23 (100.0)	1 (4.3)	4 (17.4)
orthopaedic surgeon	4 (8.5)	4 (17.4)	1 (4.3)	0 (0.0)
anaesthesiologist	29 (61.7)	17 (73.9)	1 (4.3)	1 (4.3)
radiologist	23 (48.9)	20 (87.0)	3 (13.0)	3 (13.0)
neurologist	13 (27.7)	9 (39.1)	0 (0.0)	0 (0.0)
neurosurgeon	0 (0.0)	1 (4.3)	0 (0.0)	0 (0.0)
ICU physician	2 (4.3)	5 (21.7)	0 (0.0)	0 (0.0)
Other health personnel				
anaesthesia technician	10 (21.3)	8 (34.8)	1 (4.3)	1 (4.3)
radiographer #1	35 (74.5)	20 (87.0)	4 (17.4)	12 (52.2)
radiographer #2	7 (14.9)	1 (4.3)	0 (0.0)	0 (0.0)
CT technician	8 (17.0)	6 (26.1)	1 (4.3)	1 (4.3)
laboratory staff	9 (19.1)	9 (39.1)	1 (4.3)	4 (17.4)
social worker	0 (0.0)	1 (4.3)	0 (0.0)	0 (0.0)
Other #1	3 (6.4)	6 (26.1)	1 (4.3)	3 (21.7)
Other #2	4 (8.5)	2 (8.7)	1 (4.3)	0 (0.0)
Other #3, #4 and #5	2 (4.3)	3 (13.0)	0 (0.0)	0 (0.0)

**EDs with one team n=49, two EDs with one team did not complete questionnaire (missing values for team composition)*

***EDs with a tiered response n=23: 19 of these EDs have two teams available (large and small team) and 4 of these EDs have three teams available (intermediate team next to large and small team); composition was given for all available teams and therefore this does not add up to 23*

ED = Emergency Department; ICU = Intensive Care Unit

Other #1
- One team: paediatrician (2), physician assistant;
- Tiered response, large team: paediatrician, operating room team, patient services, secretary (2), radiology backup;
- Tiered response, intermediate team: secretary;
- Tiered response, small team: paediatrician (2), secretary (2), optional specialist dependent on needs of patient.

Other #2
- One team: senior resident ICU, ICU nurse, senior resident anaesthesiology, hospital nurse coordinator (out of office hours);
- Tiered response, large team: cardiologist, paediatric intensivist (optional);
- Tiered response, intermediate team: optional specialist dependent on needs of patient.

Other #3, #4 and #5
- One team: resident radiology, resident neurology;
- Tiered response, large team: paediatric anaesthesiologist (optional), thoracic surgeon (optional), optional specialist dependent on needs of patient.
